# Encrusted Uropathy: A Comprehensive Overview—To the Bottom of the Crust

**DOI:** 10.3389/fmed.2020.609024

**Published:** 2021-01-21

**Authors:** Els Van de Perre, Gina Reichman, Deborah De Geyter, Caroline Geers, Karl M. Wissing, Emmanuel Letavernier

**Affiliations:** ^1^Nephrology Department, Universitair Ziekenhuis Brussel, Vrije Universiteit Brussel, Brussels, Belgium; ^2^Urology Department, Universitair Ziekenhuis Brussel, Vrije Universiteit Brussel, Brussels, Belgium; ^3^Microbiology Department, Universitair Ziekenhuis Brussel, Vrije Universiteit Brussel, Brussels, Belgium; ^4^Pathology Department, Universitair Ziekenhuis Brussel, Vrije Universiteit Brussel, Brussels, Belgium; ^5^Service des Explorations Fonctionnelles Multidisciplinaires, Hôpital Tenon, Assistance Publique—Hôpitaux de Paris, Sorbonne Universités, Université Pierre et Marie Curie, Paris, France; ^6^Institut National de la Santé et de la Recherche Médicale, Unité Mixte de Recherche S 1155, Paris, France

**Keywords:** encrusted pyelitis, *Corynebacterium urealyticum*, urease-producing bacteria, encrusted cystitis, encrusted uropathy

## Abstract

Encrusted uropathy is a rare subacute to chronic inflammatory disorder caused by infection with urease-producing bacteria, mainly *Corynebacterium urealyticum*. The disorder is characterized by urothelial deposition of struvite and carbonated apatite, resulting in encrustations and ulceronecrotic inflammation of the urothelium and surrounding tissues. Most commonly, encrusted uropathy is encountered in patients with predisposing conditions. The disease remains underdiagnosed. High urinary pH and negative conventional urine cultures should raise suspicion of the diagnosis. Prognosis is dependent on timely diagnosis and treatment installment, which consists of urological removal of encrustations in combination with urinary acidification and long-term antibiotic therapy.

## Introduction

Encrusted cystitis, urethritis and (uretero-)pyelitis are rare subacute to chronic inflammatory disorders caused by infection with urease-producing bacteria. These disorders are characterized by urothelial deposition of struvite and carbonated apatite, resulting in encrustations and ulceronecrotic inflammation of the urothelium and surrounding tissues. The condition was first described in 1914 by François (1), the implication of urease-producing bacteria in the pathogenesis was reported a decade later by Hager and Magath (2). Although there has been an increase in reported cases during the last decades, the condition probably remains underdiagnosed. Delayed diagnosis has potentially detrimental effects as prognosis is improved by starting treatment early in the disease process. The objective of this article is to provide a comprehensive overview of the pathogenesis, clinical presentation, diagnostic tools and treatment options in order to facilitate prompt diagnosis and correct and timely treatment installment.

### Risk Factors

Most commonly, encrusted uropathy is encountered in patients with predisposing conditions [Table 1; (3–6)], but a rare case has been described in a patient without apparent underlying risk factors (7).

**Table 1:** Predisposing conditions for the development of encrusted uropathy (non-exhaustive).

**Previous or current urological instrumentation**
- Bladder catheterization (long-term and short-term) - Ureteral catheterization, ureteral stenting - Nephrostomy
**History of endoscopic or surgical urological procedure**
- Cystoscopy, cystography - Ureterorenoscopy - Transurethral resection of the prostate - Prostatectomy - Cystectomy - Ureteral diversion - Nephrectomy
**Past or current urological disease**
- Congenital uropathy, including ectopic kidney - Neurogenic bladder dysfunction - Urolithiasis - Benign prostatic hyperplasia - Ureteropelvic junction obstruction, ureteral stenosis - Urothelial inflammation
- Radiation cystitis - History of intravesical mitomycin, adriamycin or Bacillus Calmette-Guérin (BCG) instillations - Drug-induced cystitis - Leukoplakia/malakoplakia
- Urothelial malignancy and benign neoplasms
**History of urinary tract infections (during previous 2 months)**
**Chronic debilitating disease**
- Diabetes mellitus - Liver cirrhosis - Chronic bronchitis - Cardiomyopathy - Chronic renal insufficiency - Neurological and other incapacitating disease
**Immunosuppressed status**
- Kidney transplant recipients - Immunosuppressive therapy for auto-immune disease or chronic pulmonary obstructive disease - Hematological or solid malignancy - Human Immunodeficiency Virus infection
**Long-term hospitalization (≥1 week, during previous 3 months)**
**Recent (during previous 3 months) antibiotic therapy**

While encrusted cystitis was first described in 1914 (1), the first cases of encrusted pyelitis were reported only 80 years later, in renal transplant recipients (8). These patients are more frequently affected than the general population because they often present a combination of predisposing factors comprising immunosuppressed status, long-term hospitalization, frequent antibiotic treatment, bladder and ureteral catheterization and a history of urological procedures, which may have led to the formation of fistulas, lymphoceles, and urethral or ureteral damage or stenosis. Particularly surgical reinterventions and long-term (>1 month) vesical and ureteral catheterization have been identified as important risk factors for the development of encrusted uropathy in this population (9, 10).

### Epidemiology

The incidence of encrusted pyelitis (EP) and encrusted cystitis (EC) in renal transplant recipients is estimated at 0.26–2.13% and 0.61%, respectively (9, 11), the time between renal transplantation and the diagnosis of encrusted uropathy ranging from 5 to 84 months with a mean of 24 months (9). Incidence rates of EC and EP in non-transplant patients are unknown. Although the condition remains rare, the number of reported cases has increased during the last three decades and is likely to augment even further due to increasing numbers of urological procedures, urological instrumentation and renal transplantation in older and more debilitated patients, as well as the increasing use of immunosuppressive therapies. Additionally, the detection and identification of *Corynebacterium urealyticum* (CU), the main causative agent of EC and EP has improved substantially, resulting in an increased reporting of CU bacteriuria, symptomatic urinary tract infection and encrusted uropathy. Actually, Sánchez-Martin et al. (5) described a 300% increase in positive CU urine cultures between 2009 and 2014.

## Etiopathogenesis

*Corynebacterium urealyticum* (previously *Corynebacterium CDC group D2*) is a gram-positive, strict aerobic, pleiomorphic, lipophilic, acid-fast, non-spore-forming, non-branching, fastidious, and multi-resistant urea-splitting rod. It is distinguished from the resembling *Corynebacterium diphtheriae* and *Corynebacterium jeikeium* due to its inability to reduce nitrates into nitrites, its asaccharolytic characteristic and its urease activity, which is highlighted by the name “urealyticum.” Although only first described as the cause of EC in 1985 (6), CU is now universally recognized as the principal cause of EC and EP. Other urease-producing bacteria reported as causative agents of encrusted uropathy are *Ureaplasma urealyticum*, some *Streptococcus (haemolyticus* and *viridans*) and *Staphylococcus* species, *Pseudomonas aeruginosa, Proteus, Escherichia coli, Corynebacterium glucuronolyticum*, and *Arcanobacterium pyogenes* (7, 12–14).

### *Corynebacterium urealyticum* Skin and Urinary Tract Colonization

CU is a skin commensal, colonizing 25% of hospitalized and 37% of institutionalized patients and is mainly detected in the groin area (15). Women are more frequently colonized than men (43 vs. 18%). In the normal population the cutaneous colonization rate is 0–12% (16–18). CU colonization and subsequent infection is hence predominantly hospital-acquired, with bacterial spread between patients by direct contact or airborn (19). One report even described a nosocomial outbreak in 15 patients (20). Two large case series reported 78–100% of CU bacteriuria cases to be hospital-acquired (3, 4), with time between hospital admission and bacteriuria ranging from 4 days to 6 months with a mean of 27 days (3). Skin colonization is hypothesized to be facilitated by the use of broad-spectrum antibiotics, after which urological procedures promote introduction of CU in the urinary tract. The bacterium has a special tropism for the urinary tract with strong adherence to the urothelium (21), urinary and nephrostomy catheters (22, 23), easy penetration and embedment in the mucosa (3) and biofilm formation (24, 25). In fact, in 71% of patients with positive CU urine culture, the same bacterial strain can be detected in inguinal skin culture (16), with identical antibiotic resistance patterns in skin and urinary isolates (16, 26). In a series of renal transplant recipients, skin colonization was reported as an independent risk factor for the development of CU bacteriuria (27). Additionally, hospitalization and its associated antibiotic use drive the development of antibiotic resistance (26).

The prevalence of CU bacteriuria depends on the population screened and the culture media used. In non-selected populations using non-selective media, prevalence rates of 0.04–0.20% have been reported, while the use of selective media increases the rate to 1.17% (28, 29). In selected populations detection rates augment even further, with a reported prevalence of 1.32% in hospitalized patients using non-selective media, compared to 4.64% using selective media (30). The highest rates have been described in renal transplant recipients: 1.8–10.0% vs. 9.8% with the use of non-selective media and selective media, respectively (9, 27). In one series, CU bacteriuria accounted for 3.8% of all positive urine cultures (4).

With men accounting for 55–76% of positive CU urine cultures (3–5, 16), CU bacteriuria has a clear male predilection, in contrast to CU skin colonization rates (15), likely reflecting the higher frequency of urological procedures and manipulations in males. The mean age of patients developing CU bacteriuria is 58–68 years (3–5, 16) and risk factors include prolonged hospitalization, reported in 73–75% of patients, previous urological disease in 50–64%, urological manipulation in 55–83% (bladder catheterization in 55–77%), previous urinary tract infection in 42–61%, immunosuppressed status in 27–41%, chronic debilitating disease in 48–52% and antibiotic use during the previous 3 months in 73–93% (3, 4, 16, 21). In a series of renal transplant recipients, other than CU skin colonization, independent risk factors for the development of CU bacteriuria were antibiotic treatment in the previous month and history of nephrostomy (27).

### *Corynebacterium urealyticum* Infections

Spontaneous eradication of CU from the urine has been reported in 35–41% of patients (3, 16), while in another series 15% of patients showed resolution after vesical catheter change (4). Fifty-two to seventy-six percent of patients with positive urine culture, however, develop symptomatic urinary tract infection, comprising acute and chronic cystitis, chronic prostatitis, and pyelonephritis (3, 4, 16, 31). A case of renal cyst infection has been described (32).

Although the bacterium mainly causes urinary tract infections, CU has also been described as the rare cause of wound and soft tissue infections (31, 33–35), osteomyelitis and orthopedic device-related infections (35, 36), bacteremia (34, 37–43), pneumonia (44), pericarditis (45), endocarditis (46), mediastinitis (41), and peritonitis (40), mainly in patients with underlying urological disease or other risk factors. While CU previously was considered as a non-pathogenic commensal, the bacterium is now clearly recognized as a cause of significant urinary tract and other infections. In fact, urinary presence of CU even if colony-forming units (CFU) < 100,000/mL should always be considered as pathological.

### Encrusted Uropathy

In 4–16% of patients with CU bacteriuria, encrusted urological disease develops (3–5, 16, 27). This occurs mainly in those with a suitable urothelial environment [“vesical ground” (29)], damaged by inflammation, malignancy, ischemia or urological instrumentation, creating an ideal territory for struvite encrustations. The time between urological instrumentation or intervention and diagnosis of encrusted urological disease can range from a few days to 3 years (18). Again, a male predominance (66–75%) has been reported in most series of encrusted uropathy (4, 9), with a mean age of 50–71 years (4, 9).

**Figure 1:**
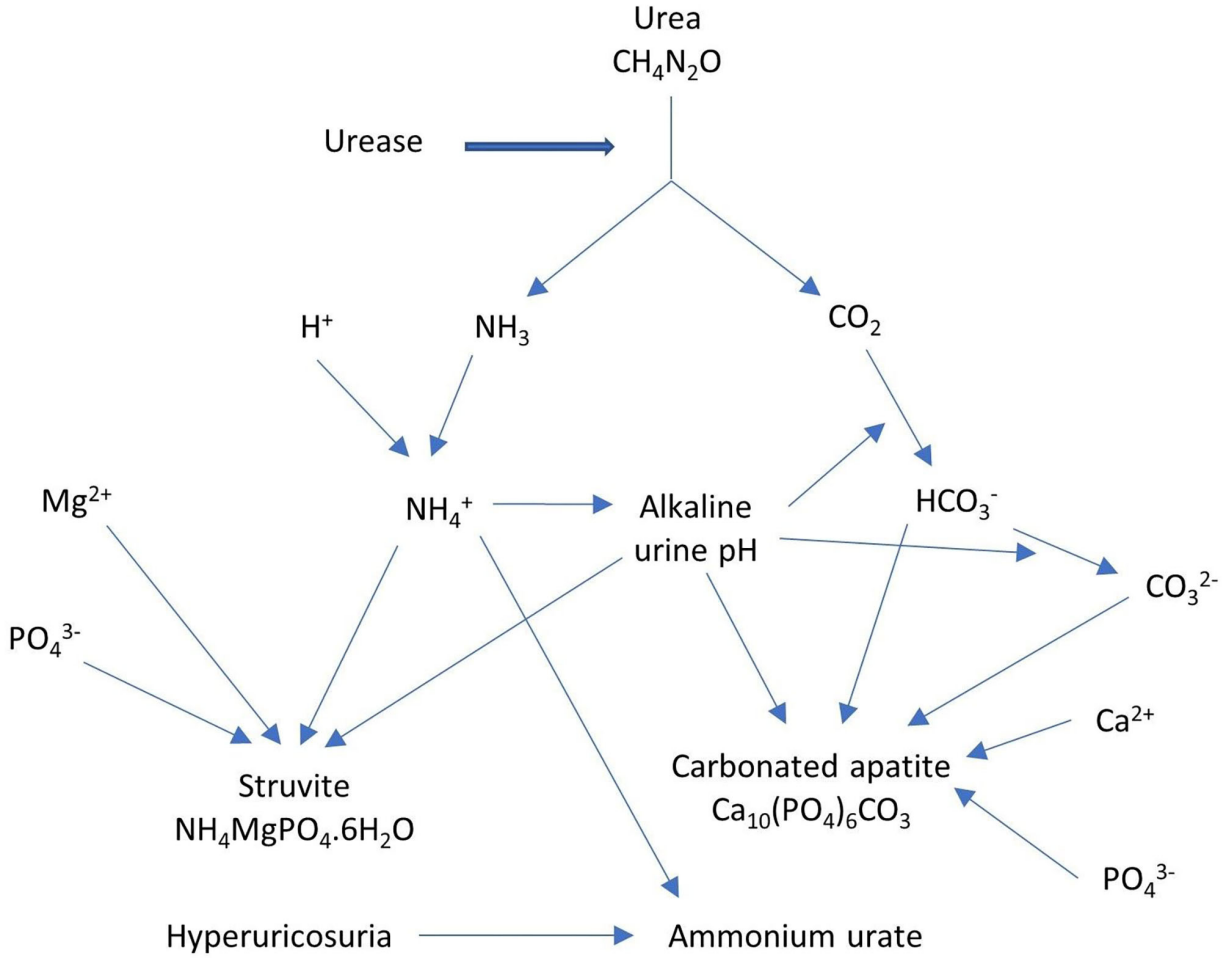
Urease as the main cause of struvite formation.

The pathogenic role in struvite (magnesium ammonium phosphate, NH_4_MgPO_4_.6H_2_O) formation has been demonstrated *in vivo* and *in vitro* for CU (47) and for other urease-producing bacteria like *Ureaplasma urealyticum* (48, 49) and *Proteus vulgaris* (47). Struvite formation is primarily caused by the bacterial urease activity (50), which generates a high urinary pH, a requisite for struvite production [Figure 1; (51)]. Elliot et al. (52) described a minimum urinary pH of 7.1 to be required for pathogenesis, although lower values have been described in some patients with encrusted uropathy (5) and struvite urolithiasis (53). Actually, urease hydrolyses urea to CO_2_ and ammonia (NH_3_), resulting in alkaline urine due to its binding with H^+^ ions resulting in the formation of ammonium (${\text{NH}}_{4}^{+}$). The high urinary pH favors the conversion of CO_2_ to bicarbonate with subsequently carbonate formation and facilitates the supersaturation of magnesium ammonium phosphate and carbonated apatite [carbapatite, Ca_10_(PO_4_)_6_CO_3_], leading to struvite and carbonated apatite crystal formation, which can cause free stone formation and/or urothelial encrustations. In the presence of hyperuricosuria, ammonium urate can be formed as well. Increased urinary pH, increased ammonium concentration, decreased urea concentration and struvite crystal formation occur as quickly as 24 h after incubation of the urine with CU and even as fast as 7 h after incubation with *Proteus vulgaris* in experimental models (47). Additionally, the increased ammonium concentrations cause cytotoxic damage to the protective mucosal glycosaminoglycan layer, expediting strong urothelial bacterial adherence, inflammation and crystal deposition (54). As opposed to carbonated apatite, which can be formed by other lithogenic processes, the presence of struvite is pathognomonic for infection with urease-producing bacteria.

The complete genome of 2 CU strains has been sequenced (55, 56), providing insight into the pathogenicity of CU (57). Its strong urease activity might be promoted by the absence of potential transcription regulator genes at the urease gene locus, located at cluster ureABCEFGD. Biofilm formation is presumed to be regulated by surA and surB genes which encode for cell surface proteins. It is assumed that urothelial adherence occurs through a surface-anchored proteinaceous pilus, encoded by genes at the SpaDEF cluster. Additionally, the rpfC gene is implicated in the resuscitation of inert bacteria and the proliferation of non-dormant, viable bacteria. Finally, horizontal gene transfer of resistance genes located on mobile genetic elements has been reported to be the primordial cause of antibiotic multiresistance.

Strong immunohistochemical staining of the osteogenic markers osteocalcin, osteonectin and osteopontin on affected bladder tissue which disappeared after treatment, has been described in a case of EC, suggesting the involvement of an osteogenic process in the pathogenesis of encrusted uropathy (58). Additionally, the implication of osteopontin in the adhesion of calcium oxalate crystals to renal epithelial cells has been reported (59). Its potential role in urothelial struvite crystal adhesion has not yet been examined.

## Clinical Presentation and Urine Analysis

EP may develop secondary to EC or can be primary due to migration of urease-producing bacteria along a nephrostomy catheter or ureteral stent, with later extension of encrusted uropathy to the bladder. EP can be unilaterally or bilaterally and can be associated with free stones, which typically are large branched pyelocaliceal (staghorn) stones. Encrustations of the bladder mucosa may lead to the development of a fibrotic, retractile bladder with impaired function and reduced bladder volume. Prostatic involvement and encrustation of foreign objects like vesical and ureteral catheters/stents and meshes has been described (5). Urethral encrustations due to encrusted urethritis may cause meatus or other urethral stenosis (60). Other causes of obstructive uropathy with unilateral or bilateral ureterohydronephrosis comprise encrustations of the bladder wall involving the ureteral orifice(s), encrustations of the ureter(s) or pyeloureteral junction(s), accompanying edema or urolithiasis. Renal capsule adherence and abscess formation due to chronic pyelonephritis may develop. Chronic kidney failure or graft dysfunction secondary to obstructive uropathy or chronic pyelonephritis, has been described in 67–86% of EP patients at diagnosis (5, 9, 61), potentially requiring renal replacement therapy.

The disease process can be both subacute, developing in <3 months, or chronic with progression for months or even years before diagnosis. Encrusted uropathy actually presents with non-specific low-grade symptoms, including dysuria or urethral discomfort, urinary frequency, stranguria, nycturia, vesical tenesmus, lumbar pain in EP and suprapubic/pelvic pain in EC (4, 9, 29). Fever is present in 25–50% of EC and in 71% of EP patients (4, 9). Macroscopic hematuria is very common, detected in 75–100% of EC and in 86% of EP patients (4, 9, 29). Urination of mucus, pus, blood, calcified or non-calcified mucopurulent debris, gravel or kidney stones can cause acute urinary retention. A strong urinary ammonia odor is frequently present (29). Sometimes general symptoms like nausea, anorexia and weight loss are associated.

Clinical examination can reveal costovertebral angle tenderness and may show encrustations at the urethral meatus (60).

Microscopic hematuria and pyuria are reported in all patients (4, 9, 29, 62). Pathogenetically required, alkaline urine pH is universal, with a pH ≥ 7.13 reported in all patients in 2 case series (9, 29) and urine pH > 8 in 89–100% of patients in 2 other series (4, 5). Alkaline urine pH is indicative for infection with urease-producing bacteria but is not specific for the diagnosis of encrusted uropathy, as urine pH >7 can be found in 62–69% of patients with CU bacteriuria without encrusted uropathy (3, 4, 27).

## Diagnosis and Differential Diagnosis

**Figure 2:**
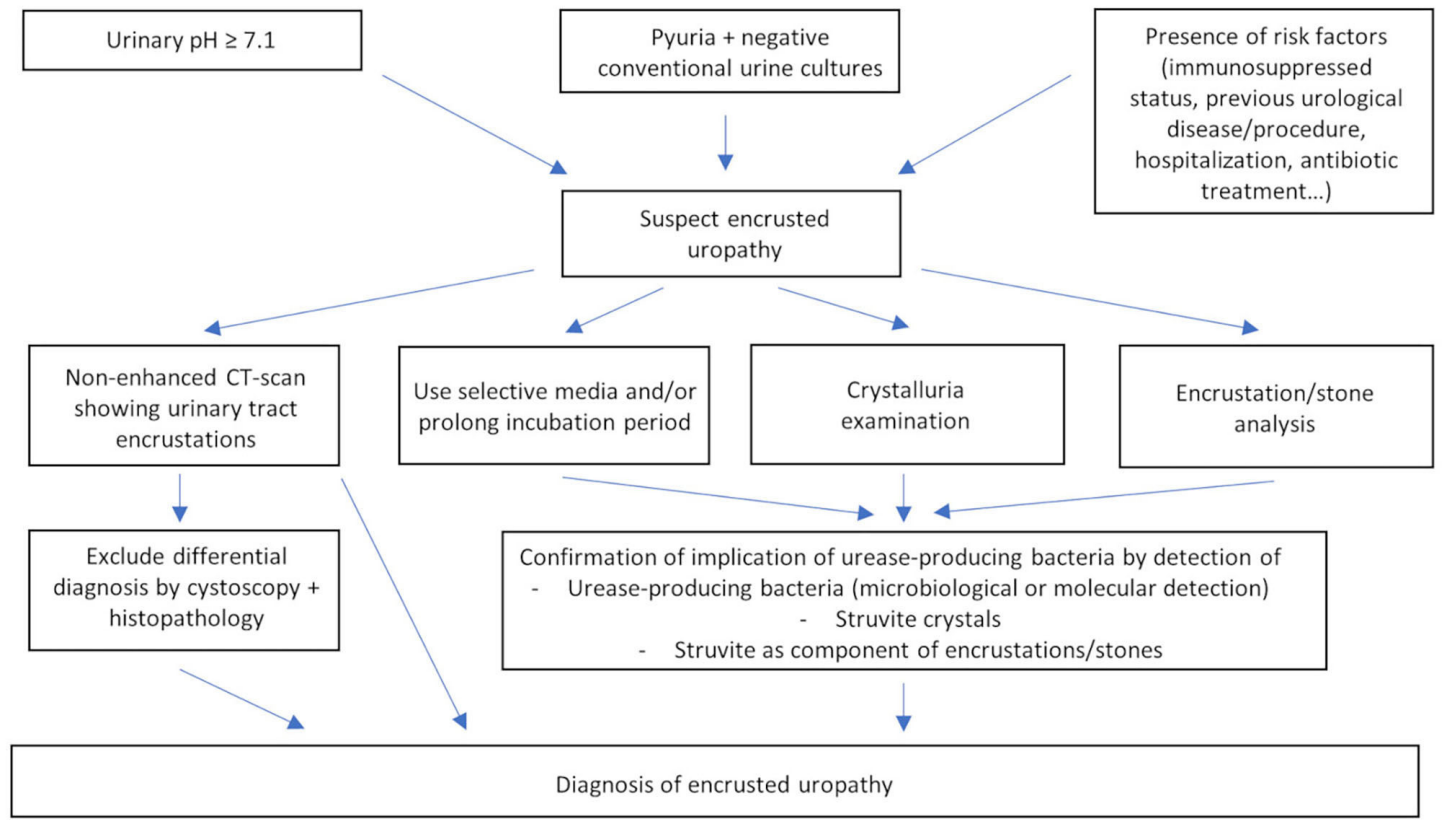
Diagnosis of encrusted uropathy.

The diagnosis can be suggested by the clinical presentation, medical history and risk factor evaluation, urine analysis, radiological and endourological findings. Confirmation of involvement of urease-producing bacteria is based on their microbiological or molecular detection in urine, bladder mucosa, encrustations or stones, on detection of struvite crystals by means of crystalluria examination or on the detection of struvite as a component of encrustations or stones (Figure 2). Additionally, other conditions in the differential diagnosis of urinary tract encrustations or calcifications need to be excluded by means of cystoscopy and histopathological examination [Table 2; (63–71)].

**Table 2:** Differential diagnosis of urinary tract calcifications/encrustations (non-exhaustive).

**Bladder wall calcifications/encrustations**
- Bladder stone - Schistosomiasis (usually submucosal calcifications, not always visible during cystoscopy, proximal extension to ureters, acidic urine) - Tuberculosis (usually submucosal calcifications, not always visible during cystoscopy, distal extension from the kidneys, frequently associated with upper urinary tract changes, acidic urine) - Amyloidosis (submucosal calcifications) - Calcified bladder tumor (adenocarcinoma, transitional cell carcinoma, squamous cell carcinoma, leiomyosarcoma, neuroblastoma, osteogenic sarcoma, pheochromocytoma, …) - Metastatic or invasive malignancy (ovarian carcinoma, coloncarcinoma, …) - Hemangioma - Hematoma - Prior radiation or photodynamic therapy - Prior cyclophosphamide treatment - Prior mitomycin C or BCG instillations - Severe infectious cystitis - Vesical malakoplakia/leukoplakia - Stevens–Johnson syndrome - Calcification of the excavated prostatic fossa - Calculus in urachal cyst - Calcified pelvic hydatid cyst - Calcified diverticulum - Echinococcus infection - Encrusted foreign body, including encrustation of intrauterine contraceptive device eroded into the bladder, stone-encrusted mesh
**Ureteral/pyelocaliceal calcifications/encrustations**
- Ureterolithiasis/pyelocaliceal stone - Schistosomiasis (usually submucosal calcifications, proximal extension from bladder, acidic urine) - Tuberculosis (usually submucosal calcifications, distal extension from the kidneys, acidic urine) - Amyloidosis (submucosal calcifications) - Ureteral argyrosis - Calcified sloughed renal papilla - Metastatic or invasive malignancy (ovarium carcinoma, coloncarcinoma, …) - Osseocartilaginous metaplasia after pelvic ureterotomy - Necrotic/calcified tumor: papilloma, urothelial or renal carcinoma, … - Encrusted foreign body

### Imaging

**Figure 3:**
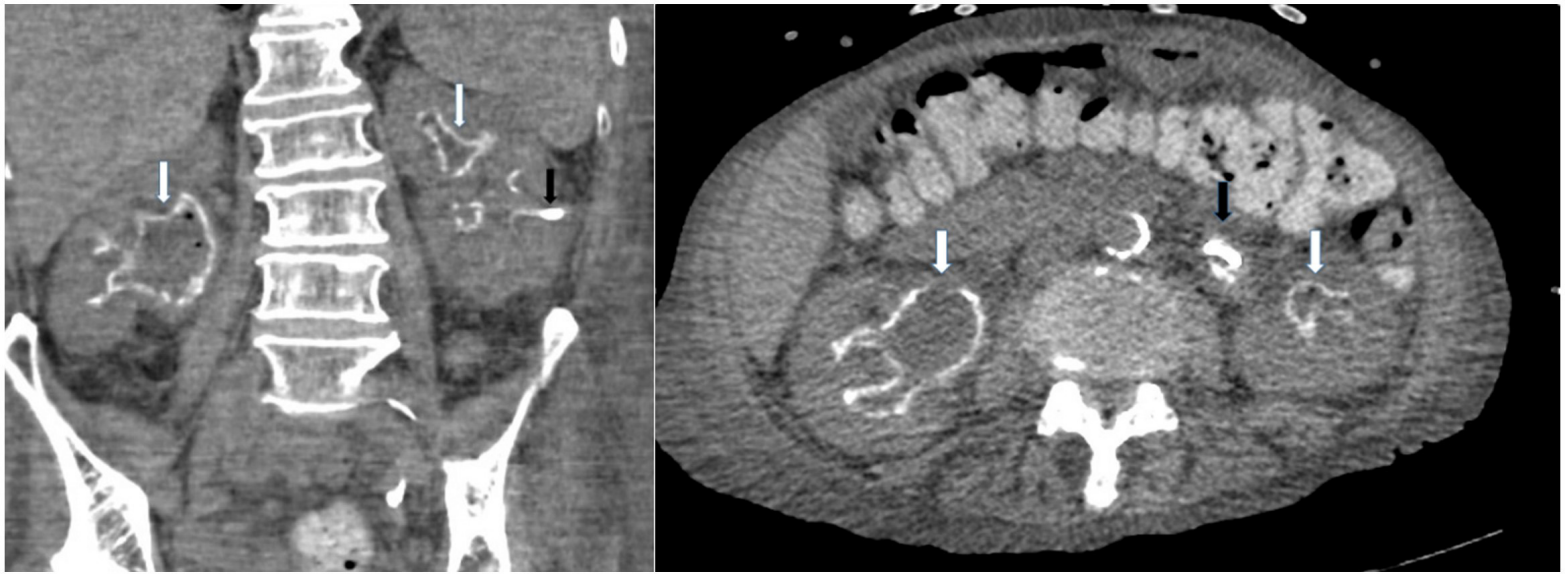
Non-enhanced CT-scan showing encrusted pyelitis. White arrow: linear calcifications of the renal collecting system with bilateral ureterohydronephrosis, black arrow: nephrostomy catheter.

Abdominal X-ray may reveal urinary tract calcifications, mainly of the bladder, but thin and occasionally radiolucent encrustations (72) are frequently missed. Accompanying staghorn calculi may be visualized. Just like for abdominal X-ray, the sensitivity for diagnosing encrusted uropathy is limited for ultrasound. In EC, thickening or irregular lining of the bladder with calcifications may be visualized while in EP hyperechogenic material in the collecting system, sometimes in association with staghorn stones, can be found. Uni- or bilateral ureterohydronephrosis can be detected. Non-enhanced CT-scan of EP shows calcifications of the renal collecting system, detected as high-density lesions, ranging from regular, thin, linear calcifications to edgy, bulky, irregular plaques. Micro-abscesses and free stones can be present. Urinary tract wall thickening, perinephric and/or periureteral inflammatory fat stranding and unilateral or bilateral ureterohydronephrosis may be demonstrated. In EC, a thickened, edematous bladder mucosa with encrustations and necrosis may be detected. Non-enhanced CT-scan has a high sensitivity and specificity and is the gold standard imaging modality for diagnosing encrusted uropathy (72) and is particularly useful for diagnosing EP (Figure 3).

### Endourology

Cystoscopic examination typically reveals a fragile, inflammatory, or hemorrhagic mucosa with ulcerations covered with white to yellow-tanned fragile encrustations. Vesical edema may impair the visualization of the ureteral orifices. The encrustations, which may resemble neoplastic lesions, may vary in size and adherence to the urothelium, ranging from small superficial fragments to large calcified encrustations deeply embedded in the bladder mucosa and are predominantly found at the trigone, ureteral orifices, bladder neck and sites of previously damaged urothelium. In EP, endourological examination can demonstrate calcified encrustations sometimes extremely closely adhered to the renal collecting system and ureters with accompanying mucosal inflammation (73). Associated staghorn stones may have a glue-like consistency (74).

### Histopathology

Macroscopically a kidney affected by EP shows clear urothelial thickening with closely adherent, superficial calcifications (72). Parenchymal abscesses can be present. In EC, the bladder mucosa can show a thin layer of fibrin mixed with calcified necrotic debris.

**Figure 4:**
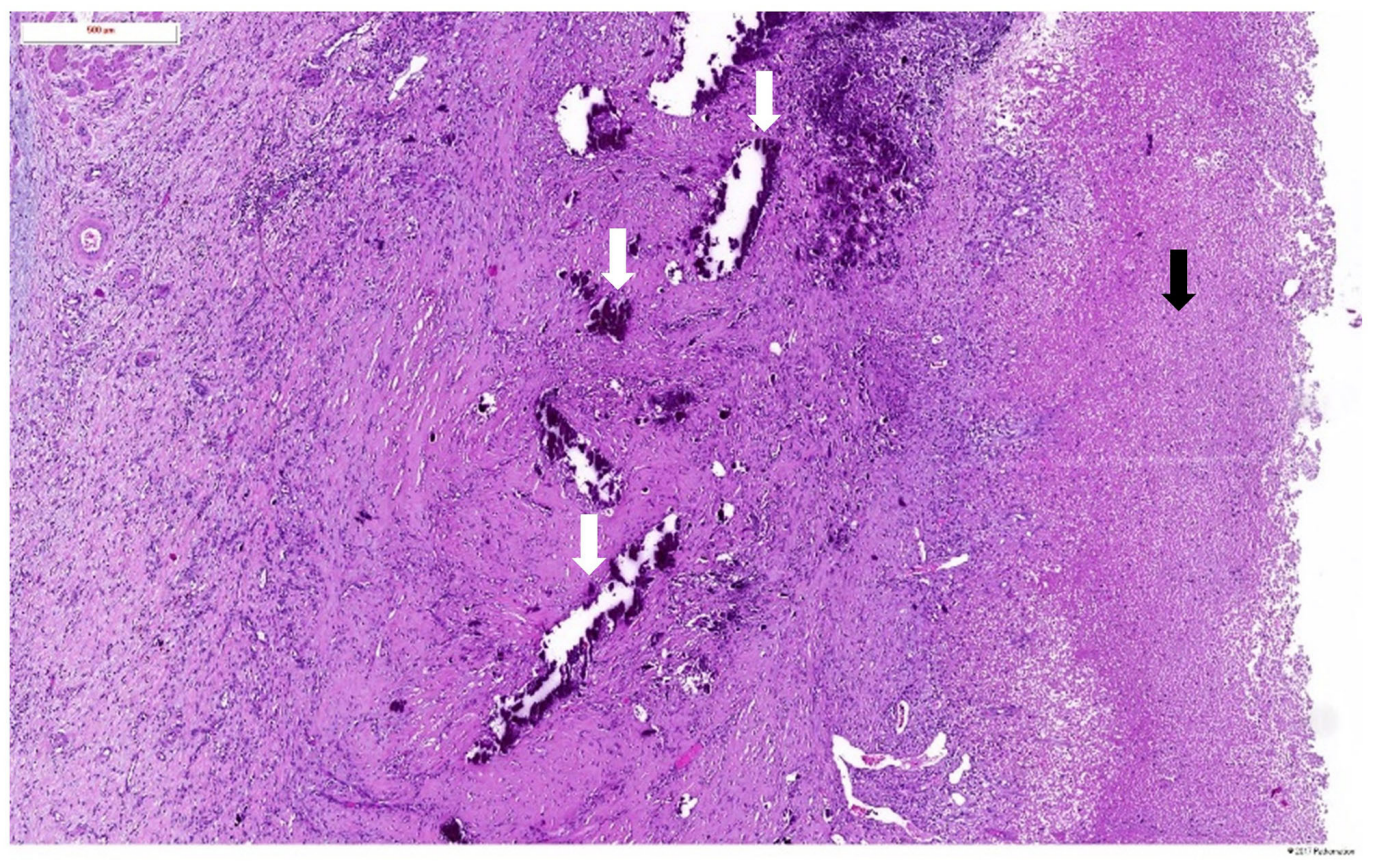
Haematoxylin and eosin staining of the pyelic mucosa in a patient with encrusted pyelitis showing a broad ulceration covered by a layer of fibrin (black arrow) and calcified necrotic debris (white arrows).

**Figure 5:**
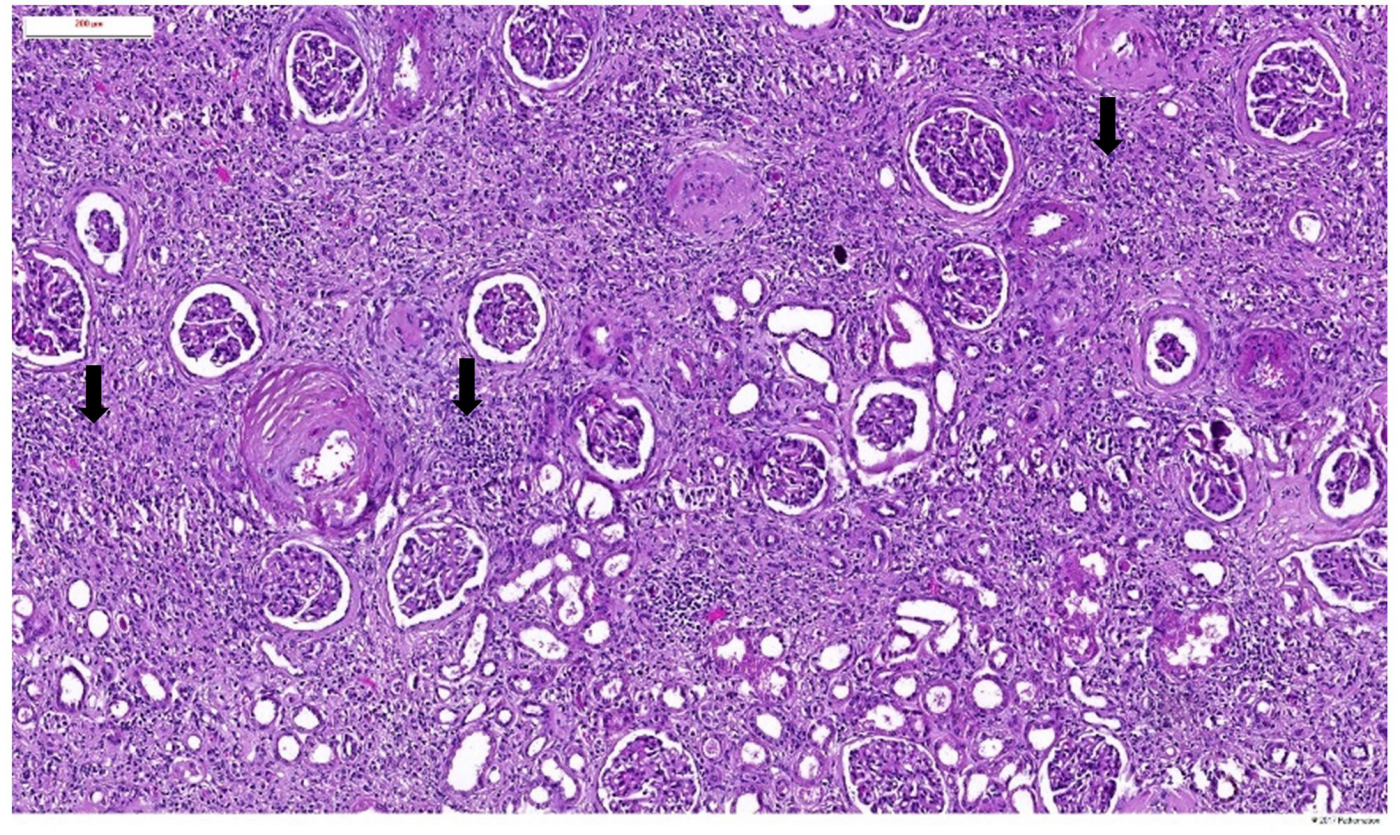
Haematoxylin and eosin staining of the renal parenchymal lesions in a patient with encrusted pyelitis showing severe chronic changes due to chronic pyelonephritis (black arrows).

Microscopic histopathological examination of tissue affected by EC/EP typically reveals 3 distinct layers (3, 9, 18). The first superficial layer consists of ulceronecrotic urothelial tissue with calcified encrustations (Figure 4) which can be demonstrated by Von Kossa stain. In zones of non-affected urothelium, there is an increased cellularity with occasionally development of degenerative lesions like squamous metaplasia. A second layer, located at the lamina propria, reveals an edematous, inflammatory infiltrate with presence of lymphocytes, plasmacytes and polymorphonuclear cells, forming a thick conglomerate, sometimes leading to granuloma formation. Occasionally eosinophils, mastocytes, fibroblasts, and histiocytes can be found. Small blood vessels are subject to thrombosis, causing necrotic areas at the superficial layer, creating a nidus for crystal deposition. Ischemia and inflammation also trigger neovascularization with increased vascular proliferation. Microabcesses with bacterial microcolonies can be present. The third and most peripheral layer corresponds to the muscularis. It usually has normal histological findings but can be the site of secondary fibrotic changes. There is very scarce knowledge about the renal parenchymal histopathological changes caused by encrusted uropathy. Severe chronic damage due to chronic pyelonephritis may be observed (Figure 5). In one case, cast formation with severe tubular damage and interstitial nephritis was described (75).

### Microbiology

Microbiological detection of CU is difficult, as it is a slow-growing bacterium for which prolonged incubation (during 48–72 h) in 5% CO_2_ on blood agar or cysteine lactose electrolyte deficient agar or on selective, enriched media is required, while conventional urine cultures generally are discarded if negative after 24 h of incubation. Initially negative conventional urine cultures with pyuria should hence raise the suspicion of encrusted uropathy and prompt the use of enriched culture media and/or prolonged incubation time, especially if struvite crystals can be detected or if the urine is alkaline.

If selective media, enriched with antibiotics impairing the growth of normal urine flora, are used, there is a 4–31 times higher urinary CU detection rate compared to the use of non-selective media (27, 28, 30). Frequently used selective media are those enriched with fosfomycin, aztreonam, polymyxin B, and amphothericin B (27, 30), those enriched with ticarcillin, fosfomycin, cefotaxime, and 5-fluorocytosine (16, 28) or those enriched with colistin and aztreonam. Rare cases of CU bacteriuria detected by conventional media but missed on selective media in case of beta-lactam sensitive strains have been described (16, 28).

Depending on the media used, simultaneous detection of other more easily identifiable bacteria has been reported in 6–57% of CU bacteriuria cases (4, 16, 27, 30). Additionally, CU can be cultured from bladder mucosa, encrustations, debris, or kidney stones (6, 16).

The bacteria grow as small whitish, opaque, smooth, convex, circular, non-motile and non-hemolytic colonies, which could traditionally be identified as CU with biochemical methods based on its urease activity and its lipophilic and asaccharolytic qualities (76). Additionally, they are catalase-positive, oxidase-negative, and nitrate-negative. Currently, however, identification of cultured bacteria is performed by Matrix-Assisted Laser Desorption Ionization-Time of Flight Mass Spectrometry (MALDI-TOF) (77), based on the bacterial protein composition. Although improvements have been made during the last decades, culturing and correct identification of CU remains challenging. Currently, molecular techniques like polymerase chain reaction (PCR) amplification and 16S rRNA gene sequencing (78, 79), RNA polymerase beta-subunit-encoding (rpoB) gene sequencing (80) and restriction fragment length polymorphism (81) are available for its detection and identification.

### Crystalluria and Encrustation Analysis

Alternatively, the involvement of urease-producing bacteria can be confirmed by the discovery of struvite crystals during crystalluria examination, which are detected frequently in patients with encrusted uropathy (4, 9, 29, 62). The detection of struvite crystals is pathognomonic for the presence of urease-producing bacteria but not for encrusted uropathy, as 27–70% of patients with CU bacteriuria without encrusted uropathy also have detectable struvite crystals (3, 4, 16, 27). Carbonated apatite crystals can also be observed (16) but are, as opposed to struvite crystals, not pathognomonic for the presence of urease-producing bacteria.

Chemical, crystallographic, or infrared spectrophotometric analysis of encrustations or kidney stones reveals the predominant presence of struvite (30–85%) and carbonated apatite (10–35%), accompanied by some minor compounds like ammonium urate, calcium oxalate, proteins, amorphous calcium phosphate, or uric acid (16, 73, 74, 82). In an experimental model, *Ureaplasma urealyticum* infection not only produced struvite but also whitlockite crystals (Ca_9_(Mg,Fe^2+^)(PO_4_)_6_PO_3_OH) (48), a component associated with infection-associated nephrolithiasis (83), but not yet described as a compound of encrustations in human encrusted uropathy.

### Differential Diagnosis

Table 2 provides an overview of the conditions to be considered in the differential diagnosis of encrusted uropathy.

## Treatment

The treatment consists of removal of encrustations by urological interventions in combination with chemolysis by means of urinary acidification and prolonged systemic antibiotic therapy for bacterial eradication. Antibiotic treatment should always proceed urinary acidification and urological treatment.

### Antibiotic Treatment

CU is an extremely multi-resistant bacterium, with complete bacterial eradication being hampered additionally by biofilm formation (24) and viability of residual bacteria within encrustations and stones. The recommended antibiotics are the glycopeptides vancomycin and teicoplanin (17, 84–86), to which the bacterium is uniformly sensitive and whose efficacy is not influenced by the urinary pH (87). Vancomycin should be administered intravenously with recommended trough levels 25–30 mcg/mL with continuous infusion and 15–20 μg/mL with intermittent administration. The possibility of intramuscular administration of teicoplanin can ensure long-term antibiotic treatment at home and limit the duration of hospitalization. Recommended dosing for intravenous and intramuscular teicoplanin is a loading dose of 1.6 g on the first day of treatment, followed by approximately 800 mg daily for through levels >30 μg/mL. Additionally, CU is consistently sensitive to linezolid (86, 88). There is a variable activity of quinolones, with fewer resistant strains with the use of the newer quinolones, although increasing resistance has also been demonstrated with use of the latter (26, 85, 89, 90). Additionally there is a variable activity of tetracyclines (26, 84, 85) and rifampin (26, 43, 84) and a reasonable activity of fusidic acid (43, 84, 91). CU is however highly resistant to penicillins, cephalosporins, carbapenems, lincosamides, aminoglycosides, macrolides, ketolides, sulphonamides, nitrofurantoin, fosfomycin, chloramphenicol, and trimethoprim-sulfamethoxazole (24, 40, 84–86, 92).

### Urological Treatment

In order to fully eradicate the infection, the encrustations should be completely removed, which frequently requires repeated urological interventions. In EC, removal of encrustations can be performed by transurethral resection, although vesical edema may complicate the procedure. In cases of prostatic involvement repeated transurethral resection of the prostate (TURP) is often necessary. In EP, extracorporeal lithotripsy is not efficacious due to close urothelial adherence of the encrustations. For the same reason and due to fibrosis and edema, endourological treatment with fragmentation of encrustations is frequently difficult and should be performed with extreme caution, as it might be complicated by hemorrhage. Additionally, the development of ureteral stenosis might complicate an endourological approach. A surgical or percutaneous approach for the treatment of EP can be considered but again may be strenuous and complicated by renal hemorrhage (73). In cases where urological removal of encrustations is incomplete, impossible or considered too hazardous, a conservative approach can be considered by means of urinary acidification in combination with prolonged antibiotic therapy (61).

### Urinary Acidification

Urinary acidification removes residual encrustations and inhibits further encrustation formation by preventing struvite and carbonated apatite supersaturation. At urinary pH < 5.5 the solubility of struvite increases significantly (93). Acidification of the urine can be performed by oral compounds or local acidification solutions. Many oral acidification formulas have been proposed and tested in experimental models (48, 94–103), but the most efficacious and the most commonly used compound in clinical practice is acetohydroxamic acid. With a molecular structure resembling urea, acetohydroxamic acid irreversibly inhibits urease, leading to increased urea concentration, reduced ammonia concentration and reduced urinary pH, hence preventing struvite crystal formation (104). Additionally, some minor bacteriostatic activity has been described (87, 104). In encrusted uropathy the available evidence is limited to case reports and case series, but in infection-induced nephrolithiasis, the efficacy of acetohydroxamic acid on stone growth reduction has been demonstrated in three randomized double-blind placebo-controlled trials (105–107). Adverse events, however, develop frequently, in 45–78% of patients, requiring cessation of therapy in 10–22% (105–107). The most frequently encountered side effects are nausea, psychoneurological symptoms like tremor and headache and musculocutaneous symptoms including myalgia, leg swelling, rash and alopecia, which all resolve upon dose reduction. Additionally, thrombophlebitis and haemolytic anemia, reversible upon temporary withdrawal of the therapy and occurring more frequently with daily dosage ≥1,500 mg and in patients with renal insufficiency, have been reported (105, 107, 108). For this reason, the recommended dosage of acetohydroxamic acid of 15 mg/kg should not exceed 1,000 mg daily, its use is contraindicated in severe renal insufficiency (serum creatinine > 3 mg/dL) and dosage reduction should be implemented in mild and moderate renal insufficiency. Close monitoring of all patients is highly recommended. Additionally, the compound is proven to be teratogenic in animals (109, 110), requiring effective contraception in females of childbearing age. Alternatively, although less effective, propionohydroxamic acid (111, 112), ammonium chloride (6, 113), vitamin C (11), cranberry juice (75), and hydroxyurea (114) have been used as oral acidifying agents. Finally, the effectiveness of l-methionine for long-term oral urinary acidification has very recently been reported in one case of encrusted uropathy by Sabiote et al. (115).

Oral urinary acidification can be sufficient in cases of limited and thin encrustations. If encrustations are extensive, additional topical acidification is necessary, especially at the start of treatment. Many solutions have been used (5, 6, 11, 18, 116–118), sometimes in association with topical antibiotics (3, 5). The use of 10% hemiacidrin solution has been prohibited by the FDA in the past after the reports of 6 deaths, probably due to urosepsis. Currently, the most frequently used solutions are Suby-G solution (citric acid 32.3 g, sodium carbonate 4.4 g, magnesium oxide 3.8 g, distilled water 1,000 mL) and Thomas' C24 solution (sodium gluconate 27 g, citric acid 27 g, malic acid 27 g, distilled water 1,000 mL) (18). Besides their acidifying capacities (the approximate pH of these solutions is 4), which increase the solubility of struvite, these solutions have bactericidal properties and can induce calcium citrate complex formation with subsequent prevention of struvite and carbonated apatite crystal formation. Continuous bladder acidification can be performed through a 22F 3-way Foley catheter or through a suprapubic catheter with outflow through a 2-way Foley catheter in EC. In EP, irrigation with an acidifying solution can be accomplished through a percutaneous nephrostomy catheter with outflow through ureteral and bladder catheters or through a secondary nephrostomy catheter. An aseptic technique should be used to prevent infection. In order to limit pain and intraparenchymal solution diffusion, precautionary measures should be taken: free inflow and outflow of the solution should be maintained, the daily amount of applied irrigation fluid should be limited to 1–2 L and the intrapelvic pressure should be <25 cm H_2_O (61), for which the height of the irrigation fluid container in relation to the patient should be carefully determined. It is recommended to start the irrigation at 10–20 mL/h and to increase the irrigation rate to a maximum of 50 mL/h according to the patient's tolerance. In general, topical acidification is well-tolerated, although pain, mild metabolic acidosis, fungal urinary tract infection, low-grade fever and pelvic edema can develop (61, 73). Additionally, there is a risk of hypermagnesemia due to Suby-G application. A recent case reported the use of intravesical dimethylsulfoxide, a weak acid with anti-inflammatory action which has been approved for interstitial cystitis and painful bladder syndrome, as add-on to transurethral removal of encrustations in the treatment of encrusted cystitis (119).

### Duration of Therapy

The optimal duration of treatment is not well established but depends on the severity of the encrustations and the condition's evolution under treatment. Mostly several weeks to a few months of treatment is necessary. Urological treatment of encrustations and urinary acidification should always be preceded by antibiotic treatment. Oral acidification can be sufficient in case of thin or few residual encrustations, while topical acidification is necessary in case of large encrustations, especially in the beginning of the treatment, which later can be switched to oral acidification when only small encrustations are remaining. In case of renal insufficiency with serum creatinine > 3 mg/dL, oral acidification is contraindicated. Monitoring of the efficacy of treatment and treatment duration determination is performed by repeated CT-scan, urine cultures, urinary pH testing and crystalluria examination. Treatment with antibiotics and urinary acidification should continue until there is complete resolution of encrustations on imaging, disappearance of struvite crystals, normalization of urine pH and sterilization of the urine.

## Outcome

Data regarding the outcome of encrusted uropathy are scarce, limited to case reports or case series and are likely biased by the selective reporting of successful cases.

The diagnosis of encrusted uropathy is frequently tardive resulting in delayed treatment installment, in which case the disease can lead to permanent obstructive nephropathy requiring indefinite nephrostomy or other urinary diversion (9, 73, 120, 121), cystectomy (122), or graft loss (9). The condition can be fatal, with mortality due to renal failure (122, 123) or sepsis due to CU or other superinfecting bacteria (124, 125). In case of correct and timely diagnosis and treatment installment, complete cure is frequent in EC (6, 9) but more difficult to obtain in EP. Sánchez-Martin et al. (5) described clinical and radiological improvement in 56 and 72% of patients with encrusted uropathy, respectively, with combination treatment, including urological removal of encrustations. Using conservative treatment, complete resolution of EP is possible (11, 73). Meria et al. (61) described complete resolution in two adult patients and nearly complete resolution in two others. Treatment can improve renal function in at least half of patients with encrusted uropathy (5, 61). Even with correct treatment installment, however, ureteral diversion or reimplantation or other intervention like (partial) cystectomy, prostatectomy, nephrectomy of graft removal can be necessary (5, 11, 61). Direct mortality due to encrusted uropathy despite correct treatment is rather infrequent but cardiovascular mortality (126) and mortality due to operative complications (73) have been described.

## Conclusion

The incidence of encrusted uropathy, although still a rare condition, is increasing. Nevertheless, the disease is probably underdiagnosed due to its non-specific clinical presentation, the relative unawareness of physicians taking care of these patients and the difficult microbiological detection and identification of *Corynebacterium urealyticum*, the main causative agent. Encrusted uropathy should be suspected in patients with underlying risk factors presenting with pyuria where conventional urine cultures remain negative, especially if urine is alkaline and when struvite crystals are present, which should prompt the use of selective culture media and/or prolong the incubation period. Non-enhanced CT-scan is the gold standard imaging modality. Prognosis is dependent on timely diagnosis and correct treatment installment, which comprises urological removal of encrustations if possible, in combination with urinary acidification and long-term antibiotic treatment.

## Author Contributions

EVdP prepared the manuscript. GR, DDG, CG, KMW, and EL reviewed and edited the manuscript. CG provided the histopathological figures. All authors contributed to the article and approved the submitted version.

## Conflict of Interest

The authors declare that the research was conducted in the absence of any commercial or financial relationships that could be construed as a potential conflict of interest.
